# A Microring Resonator Based Negative Permeability Metamaterial Sensor

**DOI:** 10.3390/s110808060

**Published:** 2011-08-17

**Authors:** Jun Sun, Ming Huang, Jing-Jing Yang, Ting-Hua Li, Yao-Zhong Lan

**Affiliations:** 1 School of Information Science and Engineering, Yunnan University, Kunming 650091, China; E-Mails: em.junsun@gmail.com (J.S.); yangjingjing@ynu.edu.cn (J.-J.Y.); litinghua_ynu@yahoo.cn (T.-H.L.); yaozhonglan_ynu@yahoo.cn (Y.-Z.L.); 2 Faculty of Metallurgical and Energy Engineering, Kunming University of Science and Technology, Kunming 650093, China

**Keywords:** metamaterials, sensor, WGMs, microring resonator

## Abstract

Metamaterials are artificial multifunctional materials that acquire their material properties from their structure, rather than inheriting them directly from the materials they are composed of, and they may provide novel tools to significantly enhance the sensitivity and resolution of sensors. In this paper, we derive the dispersion relation of a cylindrical dielectric waveguide loaded on a negative permeability metamaterial (NPM) layer, and compute the resonant frequencies and electric field distribution of the corresponding Whispering-Gallery-Modes (WGMs). The theoretical resonant frequency and electric field distribution results are in good agreement with the full wave simulation results. We show that the NPM sensor based on a microring resonator possesses higher sensitivity than the traditional microring sensor since with the evanescent wave amplification and the increase of NPM layer thickness, the sensitivity will be greatly increased. This may open a door for designing sensors with specified sensitivity.

## 1. Introduction

Due to their intriguing electromagnetic properties, a great deal of attention has been focused recently on metamaterials. The permittivity and permeability of metamaterials can be designed to continuously change from negative to positive values. Many novel metamaterial-based applications have been proposed, such as perfect lenses, cloaks, concentrators, directive antennas, superscatterers, superabsorbers, transparent devices, *etc.* [1–6]. Recently, great interest has been devoted to the sensing applications of metamaterials. For example, Jakšić *et al.* [7] investigated some peculiarities of electromagnetic metamaterials convenient for plasmon-based chemical sensing with enhanced sensitivity, and they envisioned practical applications of metamaterial-based sensors in biosensing, chemical sensing, environmental sensing, homeland security, *etc.* He *et al.* [8], studied the resonant modes of a 2D subwavelength open resonator, and showed it was suitable for biosensing. Melik *et al.* [9] presented telemetric sensing of surface strains on different industrial materials using split-ring-resonator based metamaterials, and desirable properties were obtained. Lee *et al.* [10] demonstrated experimentally the effectiveness of a split-ring resonator (SRR) array as a biosensing device at microwave frequencies. Cubukcu *et al.* [11] reported a surface enhanced molecular detection technique with zeptomole sensitivity that relies on the resonant electromagnetic coupling between a split ring resonator and the infrared vibrational modes of molecules. Alù *et al.* [12] proposed a method of dielectric sensing using *ε* near-zero narrow waveguide channels. Shreiber *et al.* [13] developed a novel microwave nondestructive evaluation sensor using a metamaterial lens for detection of material defects small relative to a wavelength. Zheludev [14] reviewed the road ahead for metamaterials, and pointed out that sensor applications are another growth area in metamaterials research. Our team has studied the performance of metamaterial sensors, and shown that the sensitivity and resolution of sensors can be greatly enhanced by using metamaterials [15–17].

WGM is a morphology-dependent resonance, which occurs when light within a dielectric microsphere, microdisk, or microring has a higher refractive index than its surroundings. In a ring resonator, WGMs form due to the total internal reflection of the light along the curved boundary surface [18]. The WGM resonance phenomenon has attracted increasing attention due to its high potential for the realization of microcavity lasers [19], quantum computers [20], sensing applications [21–29], *etc.* Examples of the applications of WGM sensors include biosensing [24], nanoparticle detection [25], single-molecule detection [26], temperature measurement [27], ammonia detection [28], and TNT detection [29]. However, to the best of our knowledge, there are no reports about any NPM sensors based on microring resonators operating in WGM.

In this paper, we derive the dispersion relation of a cylindrical dielectric waveguide loaded on a NPM layer, and compute the resonant frequencies and electric field distributions of the corresponding WGMs. We perform a full wave simulation of the performance of the NPM sensor, and compared it with the theoretical results. We show that the NPM sensor possesses much higher sensitivity than a traditional microring sensor, and the mechanism behind these phenomena is verified by theoretical analysis and simulation.

## 2. Theoretical Analysis

**Figure 1.**
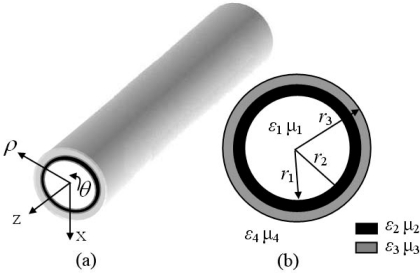
**(a)** Model of the four-layer cylindrical dielectric waveguide; **(b)** cross section of the waveguide.

Figure 1 shows the geometry of a cylindrical dielectric waveguide loaded with a layer of metamaterials. The inner side of the e cylindrical dielectric waveguide (*ε*_3_,*μ*_3_) is loaded on a metamaterial layer (*ε*_2_,*μ*_2_). The waveguide has a four-layer structure. The material parameters of regions 1, 2, 3, 4 are denoted as (*ε*_1_,*μ*_1_), (*ε*_2_,*μ*_2_), (*ε*_3_,*μ*_3_), (*ε*_4_,*μ*_4_), respectively. The axial fields in corresponding regions for TM mode [30] are:

(1a) $$E_{z}^{\left(1\right)}(r,\theta )=A_{m}J_{m}(p_{1}r)e^{\pm \mathrm{jm}\theta }$$

(1b) $$E_{z}^{\left(2\right)}(r,\theta )=(B_{m}J_{m}(p_{2}r)+B_{m}^{\prime }Y_{m}(p_{2}r))e^{\pm \mathrm{jm}\theta }$$

(1c) $$E_{z}^{\left(3\right)}(r,\theta )=(C_{m}J_{m}(p_{3}r)+C_{m}^{\prime }Y_{m}(p_{3}r))e^{\pm j\mathrm{m\theta}}$$

(1d) $$E_{z}^{\left(4\right)}(r,\theta )=D_{m}K_{m}(qr))e^{\pm \mathrm{jm}\theta }$$

where *A_m_*, *B_m_*, *C_m_*, *D_m_*, $B_{m}^{\prime }$ and ${C^{\prime }}_{m}$ are chosen here to weight the field, but they are interdependent. The functions *J_m_*, *Y_m_*, and *K_m_* are, respectively, the Bessel functions of the first kind, of the second kind, and the modified Bessel function of the second kind. The terms $p_{1}=\sqrt{\omega^{2}\varepsilon_{1}\mu_{1}-\beta^{2}}$, $p_{2}=\sqrt{\omega^{2}\varepsilon_{2}\mu_{2}-\beta^{2}}$, $p_{3}=\sqrt{\omega^{2}\varepsilon_{3}\mu_{3}-\beta^{2}}$, $q=\sqrt{\beta^{2}-\omega^{2}\varepsilon_{4}\mu_{4}}$. *β* is the propagation constant, and *m* is the angular order. For an infinite cylindrical dielectric waveguide with negligible absorption and no axial component of the propagation constant (*β* = 0), TM mode degenerates to WGM [31], and Equation (1) becomes:

(2a) $$E_{z}^{\left(1\right)}(r,\theta )=A_{m}J_{m}(p_{1}r)e^{\pm \mathrm{jm}\theta }$$

(2b) $$E_{z}^{\left(2\right)}(r,\theta )=(B_{m}J_{m}(p_{2}r)+B_{m}^{\prime }Y_{m}(p_{2}r))e^{\pm \mathrm{jm}\theta }$$

(2c) $$E_{z}^{\left(3\right)}(r,\theta )=(C_{m}J_{m}(p_{3}r)+C_{m}^{\prime }Y_{m}(p_{3}r))e^{\pm \mathrm{jm}\theta }$$

(2d) $$E_{z}^{\left(4\right)}(r,\theta )=D_{m}^{\prime }H_{m}^{\left(1\right)}(p_{4}r))e^{\pm \mathrm{jm}\theta }$$

where $p_{1}=\omega \sqrt{\varepsilon_{1}\mu_{1}}$, $p_{2}=\omega \sqrt{\varepsilon_{2}\mu_{2}}$, $p_{3}=\omega \sqrt{\varepsilon_{3}\mu_{3}}$, $p_{4}=\sqrt{-q^{2}}=\omega \sqrt{\varepsilon_{4}\mu_{4}}$, $D_{m}^{\prime }=(i\pi /2)e^{\mathrm{im}\pi /2}D_{m}$, $H_{m}^{\left(1\right)}$ is the Hankel function of the first kind. The relation between $H_{m}^{\left(1\right)}$ and *K_m_* is $K_{m}(-iz)=(i\pi /2)e^{\mathrm{im}\pi /2}H_{m}^{\left(1\right)}(z)$. For TM mode in an infinite cylindrical dielectric waveguide, transverse magnetic fields can be obtained as:

(3a) $$H_{r}(r,\theta )=\frac{1}{p^{2}}\left(\frac{j\omega \varepsilon }{r}\frac{\partial E_{z}(r,\theta )}{\partial \theta }\right)$$

(3b) $$H_{\theta }(r,\theta )=\frac{1}{p^{2}}\left(-j\omega \varepsilon \frac{\partial E_{z}(r,\theta )}{\partial r}\right)$$

The tangential fields matching equations at the boundary surfaces *r*=*r*_1_, *r*=*r*_2_ and *r*=*r*_3_ are expressed as:

$$\begin{array}{l} E_{z}^{\left(1\right)}(r_{1},\theta )=E_{z}^{\left(2\right)}(r_{1},\theta ),\;\;H_{\theta }^{\left(1\right)}(r_{1},\theta )=H_{\theta }^{\left(2\right)}(r_{1},\theta ),\;\;E_{z}^{\left(2\right)}(r_{2},\theta )=E_{z}^{\left(3\right)}(r_{2},\theta ),\\ H_{\theta }^{\left(2\right)}(r_{2},\theta )=H_{\theta }^{\left(3\right)}(r_{2},\theta ),\;\;E_{z}^{\left(3\right)}(r_{3},\theta )=E_{z}^{\left(4\right)}(r_{3},\theta ),\;\;H_{\theta }^{\left(3\right)}(r_{4},\theta )=H_{\theta }^{\left(4\right)}(r_{4},\theta ) \end{array}$$

Satisfying these conditions gives:

(4) $$[M]{\left[A_{m},B_{m},B_{m}^{\prime },C_{m},C_{m}^{\prime },D_{m}^{\prime }\right]}^{T}=0$$

where:

(5) $$[M]=\left[\begin{array}{cccccc} J_{m}(p_{1}r_{1}) & -J_{m}(p_{2}r_{1}) & -Y_{m}(p_{2}r_{1}) & 0 & 0 & 0\\ -\frac{\varepsilon_{1}J_{m}^{\prime }(p_{1}r_{1})}{p_{1}} & \frac{\varepsilon_{2}J_{m}^{\prime }(p_{2}r_{1})}{p_{2}} & \frac{\varepsilon_{2}Y_{m}^{\prime }(p_{2}r_{1})}{p_{2}} & 0 & 0 & 0\\ 0 & J_{m}(p_{2}r_{2}) & Y_{m}(p_{2}r_{2}) & -J_{m}(p_{3}r_{2}) & -Y_{m}(p_{3}r_{2}) & 0\\ 0 & -\frac{\varepsilon_{2}J_{m}^{\prime }(p_{2}r_{2})}{p_{2}} & -\frac{\varepsilon_{2}Y_{m}^{\prime }(p_{2}r_{2})}{p_{2}} & \frac{\varepsilon_{3}J_{m}^{\prime }(p_{3}r_{2})}{p_{3}} & \frac{\varepsilon_{3}Y_{m}^{\prime }(p_{3}r_{2})}{p_{3}} & 0\\ 0 & 0 & 0 & J_{m}(p_{3}r_{3}) & Y_{m}(p_{3}r_{3}) & -H_{m}^{\left(1\right)}(p_{4}r_{3})\\ 0 & 0 & 0 & -\frac{\varepsilon_{3}J_{m}^{\prime }(p_{3}r_{3})}{p_{3}} & -\frac{\varepsilon_{3}Y_{m}^{\prime }(p_{3}r_{3})}{p_{3}} & \frac{\varepsilon_{4}H_{m}^{\prime (1)}(p_{4}r_{3})}{p_{4}} \end{array}\right]$$

The dispersion equation can be obtained by setting | *M* |=0. The resonant frequency for different modes can be calculated using the software Mathematica (Wolfram Research Inc., Champaign, IL, USA). Coefficients *B_m_*, $B_{m}^{\prime }$, *C_m_*, $C_{m}^{\prime }$ and $D_{m}^{\prime }$ can be expressed in terms of the arbitrary coefficient *A_m_*, and $B_{m}=f_{m}^{\left(1\right)}A_{m}$, ${B^{\prime }}_{m}=f_{m}^{\left(2\right)}A_{m}$, $C_{m}=f_{m}^{\left(3\right)}A_{m}$, $C_{m}^{\prime }=f_{m}^{\left(4\right)}A_{m}$, $D_{m}^{\prime }=f_{m}^{\left(5\right)}A_{m}$. Parameters $f_{n}^{\left(1\right)}$, $f_{n}^{\left(2\right)}$, $f_{n}^{\left(3\right)}$, $f_{n}^{\left(4\right)}$ and $f_{n}^{\left(5\right)}$ may be found from Equation (4). Electric field distribution for different mode can be obtained by substituting these coefficients in to Equation (2):

(6) $$f_{m}^{\left(1\right)}=(p_{2}\varepsilon_{1}J_{m}^{\prime }(p_{1}r_{1})Y_{m}(p_{2}r_{1})-p_{1}\varepsilon_{2}J_{m}(p_{1}r_{1})Y_{m}^{\prime }(p_{2}r_{1}))/(p_{1}\varepsilon_{2}J_{m}^{\prime }(p_{2}r_{1})Y_{m}(p_{2}r_{1})-p_{1}\varepsilon_{2}J_{m}(p_{2}r_{1})Y_{m}^{\prime }(p_{2}r_{1}))$$

(7) $$f_{m}^{\left(2\right)}=-(p_{2}\varepsilon_{1}J_{m}^{\prime }(p_{1}r_{1})J_{m}(p_{2}r_{1})-p_{1}\varepsilon_{2}J_{m}(p_{1}r_{1})J_{m}^{\prime }(p_{2}r_{1}))/(p_{1}\varepsilon_{2}J_{m}^{\prime }(p_{2}r_{1})Y_{m}(p_{2}r_{1})-p_{1}\varepsilon_{2}J_{m}(p_{2}r_{1})Y_{m}^{\prime }(p_{2}r_{1}))$$

(8) $$\begin{array}{l} f_{m}^{\left(3\right)}=p_{2}{ɛ}_{3}Y_{m}^{\prime }(p_{3}r_{2})J_{m}(p_{2}r_{2})(p_{1}{ɛ}_{2}J_{m}(p_{1}r_{1})Y_{m}^{\prime }(p_{2}r_{1})-p_{2}{ɛ}_{1}J_{m}^{\prime }(p_{1}r_{1})Y_{m}(p_{2}r_{1}))+Y_{m}(p_{2}r_{2})\cdot \\ (p_{2}{ɛ}_{1}J_{m}^{\prime }(p_{1}r_{1})J_{m}(p_{2}r_{1})-p_{1}{ɛ}_{2}J_{m}(p_{1}r_{1})J_{m}^{\prime }(p_{2}r_{1})))+p_{3}{ɛ}_{2}Y_{m}(p_{3}r_{2})(J_{m}^{\prime }(p_{2}r_{2})(p_{2}{ɛ}_{1}J_{m}^{\prime }(p_{1}r_{1})\cdot \\ Y_{m}(p_{2}r_{1})-p_{1}{ɛ}_{2}J_{m}(p_{1}r_{1})Y_{m}^{\prime }(p_{2}r_{1}))+Y_{m}^{\prime }(p_{2}r_{2})(p_{1}{ɛ}_{2}J_{m}(p_{1}r_{1})J_{m}^{\prime }(p_{2}r_{1})-p_{2}{ɛ}_{1}J_{m}^{\prime }(p_{1}r_{1})J_{m}(p_{2}r_{1}))))\\ /(p_{1}p_{2}{ɛ}_{2}{ɛ}_{3}(J_{m}^{\prime }(p_{2}r_{1})Y_{m}(p_{2}r_{1})-J_{m}(p_{2}r_{1})Y_{m}^{\prime }(p_{2}r_{1})){J^{\prime }}_{m}(p_{3}r_{2})Y_{m}(p_{3}r_{2})-J_{m}(p_{3}r_{2})Y_{m}^{\prime }(p_{3}r_{2}))) \end{array}$$

(9) $$\begin{array}{l} f_{m}^{\left(4\right)}=p_{2}{ɛ}_{3}J_{m}^{\prime }(p_{3}r_{2})J_{m}(p_{2}r_{2})(p_{2}{ɛ}_{1}J_{m}^{\prime }(p_{1}r_{1})Y_{m}(p_{2}r_{1})-p_{1}{ɛ}_{2}J_{m}(p_{1}r_{1})Y_{m}^{\prime }(p_{2}r_{1}))+Y_{m}(p_{2}r_{2})\cdot \\ (p_{1}{ɛ}_{2}J_{m}(p_{1}r_{1})J_{m}^{\prime }(p_{2}r_{1})-p_{2}{ɛ}_{1}J_{m}^{\prime }(p_{1}r_{1})J_{m}(p_{2}r_{1})))+p_{3}{ɛ}_{2}J_{m}(p_{3}r_{2})(J_{m}^{\prime }(p_{2}r_{2})(p_{1}{ɛ}_{2}J_{m}(p_{1}r_{1})\cdot \\ Y_{m}^{\prime }(p_{2}r_{1})-p_{2}{ɛ}_{1}J_{m}^{\prime }(p_{1}r_{1})Y_{m}(p_{2}r_{1}))+Y_{m}^{\prime }(p_{2}r_{2})(p_{2}{ɛ}_{1}J_{m}^{\prime }(p_{1}r_{1})J_{m}(p_{2}r_{1})-p_{1}{ɛ}_{2}J_{m}(p_{1}r_{1})J_{m}^{\prime }(p_{2}r_{1}))))\\ /(p_{1}p_{2}{ɛ}_{2}{ɛ}_{3}(J_{m}^{\prime }(p_{2}r_{1})Y_{m}(p_{2}r_{1})-J_{m}(p_{2}r_{1})Y_{m}^{\prime }(p_{2}r_{1}))J_{m}^{\prime }(p_{3}r_{2})Y_{m}(p_{3}r_{2})-J_{m}(p_{3}r_{2})Y_{m}^{\prime }(p_{3}r_{2}))) \end{array}$$

(10) $$\begin{array}{c} f_{m}^{\left(5\right)}=(Y_{m}(p_{3}r_{3})(p_{2}{ɛ}_{3}J_{m}^{\prime }(p_{3}r_{2})(J_{m}(p_{2}r_{2})(p_{2}{ɛ}_{1}J_{m}^{\prime }(p_{1}r_{1})Y_{m}(p_{2}r_{1})-p_{1}{ɛ}_{2}J_{m}(p_{1}r_{1})Y_{m}^{\prime }(p_{2}r_{1}))\\ +Y_{m}(p_{2}r_{2})(p_{1}{ɛ}_{2}J_{m}(p_{1}r_{1})J_{m}^{\prime }(p_{2}r_{1})-p_{2}{ɛ}_{1}J_{m}^{\prime }(p_{1}r_{1})J_{m}(p_{2}r_{1})))+p_{3}{ɛ}_{2}J_{m}(p_{3}r_{2})(J_{m}^{\prime }(p_{2}r_{2})(p_{1}{ɛ}_{2}J_{m}(p_{1}r_{1})\cdot \\ Y_{m}^{\prime }(p_{2}r_{1})-p_{2}{ɛ}_{1}J_{m}^{\prime }(p_{1}r_{1})Y_{m}(p_{2}r_{1}))+Y_{m}^{\prime }(p_{2}r_{2})(p_{2}{ɛ}_{1}J_{m}^{\prime }(p_{1}r_{1})J_{m}(p_{2}r_{1})-p_{1}{ɛ}_{2}J_{m}(p_{1}r_{1})J_{m}^{\prime }(p_{2}r_{1}))))+\\ J_{m}(p_{3}r_{3})(p_{2}{ɛ}_{3}Y_{m}^{\prime }(p_{3}r_{2})(J_{m}(p_{2}r_{2})(p_{1}{ɛ}_{2}J_{m}(p_{1}r_{1})Y_{m}^{\prime }(p_{2}r_{1})-p_{2}{ɛ}_{1}J_{m}^{\prime }(p_{1}r_{1})Y_{m}(p_{2}r_{1}))+Y_{m}(p_{2}r_{2})\cdot \\ \left(p_{2}{ɛ}_{1}J_{m}^{\prime }(p_{1}r_{1})J_{m}(p_{2}r_{1}))-p_{1}{ɛ}_{2}J_{m}(p_{1}r_{1})J_{m}^{\prime }(p_{2}r_{1})))+p_{3}{ɛ}_{2}Y_{m}(p_{3}r_{2})(J_{m}^{\prime }(p_{2}r_{2})(p_{2}{ɛ}_{1}J_{m}^{\prime }(p_{1}r_{1})Y_{m}(p_{2}r_{1}\right)\\ -p_{1}{ɛ}_{2}J_{m}(p_{1}r_{1})Y_{m}^{\prime }(p_{2}r_{1}))+Y_{m}^{\prime }(p_{2}r_{2})(p_{1}{ɛ}_{2}J_{m}(p_{1}r_{1})J_{m}^{\prime }(p_{2}r_{1})-p_{2}{ɛ}_{1}J_{m}^{\prime }(p_{1}r_{1})J_{m}(p_{2}r_{1})))))\\ /(p_{1}p_{2}{ɛ}_{2}{ɛ}_{3}H_{m}^{\left(1\right)}(p_{4}r_{3})(J_{m}^{\prime }(p_{2}r_{1})Y_{m}(p_{2}r_{1})-J_{m}(p_{2}r_{1})Y_{m}^{\prime }(p_{2}r_{1}))(J_{m}^{\prime }(p_{3}r_{2})Y_{m}(p_{3}r_{2})-J_{m}(p_{3}r_{2})Y_{m}^{\prime }(p_{3}r_{2}))) \end{array}$$

## 3. Results and Discussion

**Figure 2.**
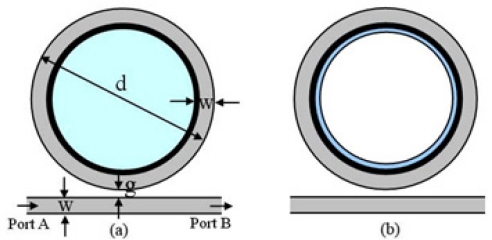
Simulation model of the NPM sensor: **(a)** homogeneous sensing; **(b)** surface sensing.

Simulation models of the NPM sensor based on a microring resonator are shown in Figure 2. A layer of NPM with thickness *t* is located on the inner side of the microring. Permittivity and permeability of the NPM are *ε*_2_ = *ε*_0_, *μ*_2_ = –*μ*_0_. Width of the microring and the waveguide is *w* = 0.3 μm. The outer diameter of the microring is *d* = 5 μm. The distance from outer microring to the waveguide is *g* = 0.232 μm. The permittivity of the microring and the waveguide is *ε*_3_ = 10.24*ε*_0_. Figure 2(a) is the simulation model for homogeneous sensing. The dielectric core with permittivity *ε*_1_ = *ε_r_ε*_0_ is colored in light blue. Figure 2(b) is the simulation model for surface sensing. The dielectric substance with thickness *t_s_* and permittivity *ε*_1_ = *ε_r_ε*_0_ is attached to the NPM layer.

**Figure 3.**
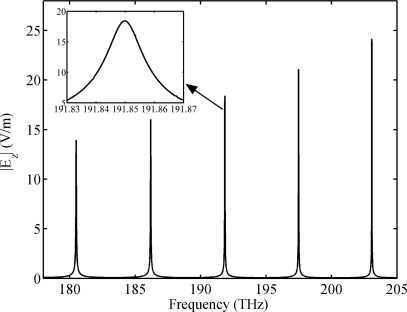
Frequency spectrum of the NPM sensor. Thickness of the NPM layer is *t* = 0.09 *μ*m. Permittivity of the dielectric core is *ε_r_* = 1.

The frequency spectrum of the NPM sensor for homogeneous sensing is simulated by the finite element software COMSOL Multiphysics (COMSOL Inc., Burlington, MA, USA), as shown in Figure 3. In the simulation, the computational space is surrounded by a scattering boundary. The excitation is set at port A of the waveguide. The spectrum is obtained by frequency sweep. From left to right, the spectral lines represent modes 25, 26, 27, 28 and 29 of the NPM sensor. The inset shows the amplification in the 191.83–191.87 THz frequency range. Table 1 shows the comparison of the analytical and simulated resonant frequency for the microring sensor and the NPM sensor. Therefore, WGMs (*m* = 25, 26, 27, 28, 29) in the cross section of the waveguide correspond to the modes of the microring sensor and the NPM sensor. The analytical resonant frequency of the sensor can be obtained by setting | *M* | = 0 (details may be found in next Section). The maximum deviation between simulation results and analytical results is 0.011 THz. The analytical results are in good agreement with the simulation results.

**Figure 4.**
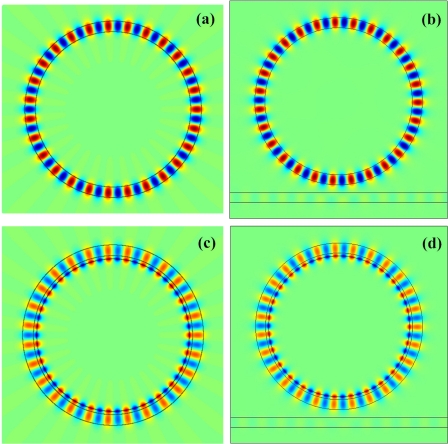
Electric field distribution of the WGM operating at mode 27. **(a)** The cross section of the waveguide; **(b)** the microring resonator; **(c)** the cross section of the waveguide loaded on NPM layer; **(d)** the microring resonator loaded on NPM layer. Thickness of the NPM layer is *t* = 0.09 *μ*m.

Supposed that material parameters of the waveguide in region 1, 2, 3, 4 are *ε*_1_ = *ε_r_ε*_0_, *μ*_1_ = *μ*_0_, *ε*_2_ = *ε*_0_, *μ*_2_ = –*μ*_0_, *ε*_3_ = 10.24*ε*_0_, *μ*_3_ = *μ*_0_, *ε*_4_ = *ε*_0_, *μ*_4_ = *μ*_0_, respectively. The resonant frequency of WGM (*m* = 27) in the cross section of the waveguide can be calculated by setting | *M* |=0. The coefficients $B_{m}=f_{m}^{\left(1\right)}A_{m}$, $B_{m}^{\prime }=f_{m}^{\left(2\right)}A_{m}$, $C_{m}=f_{m}^{\left(3\right)}A_{m}$, $C_{m}^{\prime }=f_{m}^{\left(4\right)}A_{m}$, $D_{m}^{\prime }=f_{m}^{\left(5\right)}A_{m}$ can be easily obtained according to Equations (6)–(10). The electric field distribution of the WGM can be calculated according to Equation (2), and are shown in Figure 4(a,c). To confirm the WGM in the cross section of the waveguide corresponds to the mode of the microring resonator, we simulate the electric field distribution of the microring resonator, as shown in Figure 4(b,d). From Figure 4, we can observe that the theoretical results are in good agreement with the simulation results. Interestingly, we find that the maximum electric field is located at the interface of the NPM layer and core medium. This implies that a microring resonator loaded on an NPM layer has higher sensitivity than a traditional microring resonator without loading on the NPM layer.

**Figure 5.**
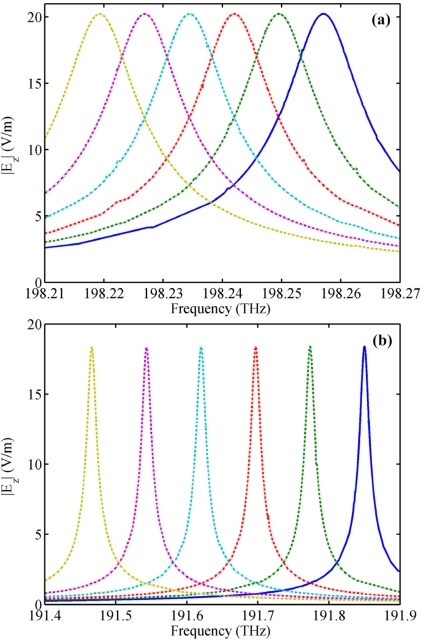
Resonant frequency spectrum of mode 27 with respect to the change of core medium permittivity *ε_r_*. From left to right, the curves correspond to *ε_r_* = 1, 1.02, 1.04, 1.06, 1.08 and 1.1, respectively. **(a)** The microring sensor; **(b)** the NPM sensor. Thickness of the NPM layer is *t* = 0.09 μm.

**Figure 6.**
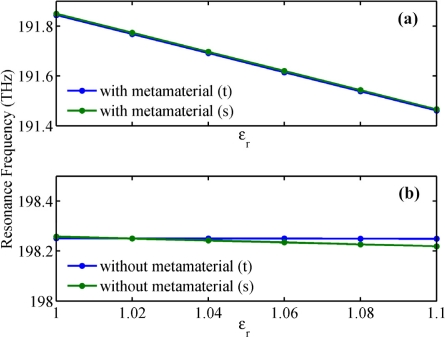
Relation between *ε_r_* and resonant frequency. **(a)** NPM sensor; **(b)** Microring sensor.

To confirm the above idea, we simulated the performance of the microring sensor and the NPM sensor for homogeneous sensing, as shown in Figure 5. Permittivity (*ε_r_*) of the dielectric core varies from 1 to 1.1 with an interval of 0.02. From Figure 5(a,b), we can observe that the spectra red shift with the increase of *ε_r_*. Sensitivity for the microring sensor and the NPM sensor is 5.9 nm/RIU and 64.2 nm/RIU, respectively. Here, sensitivity is defined as $\Delta \lambda /\Delta n=[\lambda (\varepsilon_{r},t)-\lambda (1,t)]/(\sqrt{\varepsilon_{r}}-1)$. Figure 6(a,b) show the analytical and simulating resonant frequency for the NPM sensor and microring sensor, respectively. Simulating frequencies are calculated from Figure 5, while the theoretical frequencies are obtained by setting | *M* |=0. From Figure 6, we find that the simulation results are in good agreement with the theoretical results. With an increase of 0.02 in core medium permittivity, average frequency shift for the NPM sensor is very large [Figure 6(a)], but the average frequency shift of the microring sensor is quite small [Figure 6(b)]. Therefore, the NPM sensor possesses much higher sensitivity than the traditional microring sensor.

**Figure 7.**
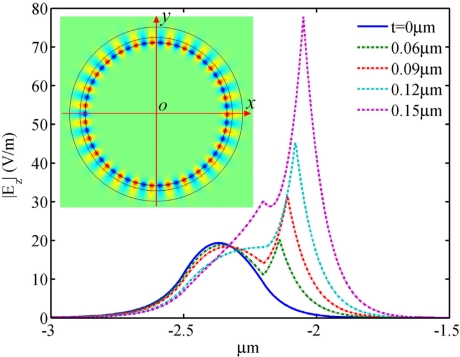
Electric field distribution along *x* axis from −3 μm to −1.5 μm for the NPM sensor operating in mode 27. The inset shows the electric field distribution of the NPM sensor, of which the NPM layer thickness is *t* = 0.15 μm.

**Figure 8.**
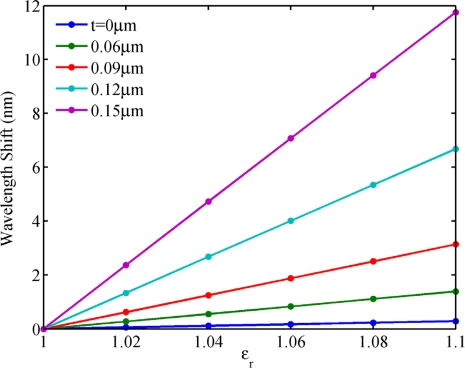
Homogeneous sensing. Relation between *ε_r_* and wavelength shift for a variation of NPM layer thickness.

To reveal the mechanism behind these phenomena, we plotted the electric field distribution of the NPM sensor along the *x* axis from −3 μm to −1.5 μm for mode 27, as shown in Figure 7. Permittivity of the core medium is set to be *ε_r_* = 1. It is seen that the electric field intensity increases with NPM layer thickness (*t*). The inset shows the electric field distribution of the NPM sensor. From Figure 7, we can clearly observe that the stronger electric field of evanescent wave penetrates into the detecting region when the thickness of NPM layer increases. Figure 8 shows the relation between core medium permittivity and wavelength shift for different NPM layer thickness. Permittivity of the core medium increases from 1 to 1.1 with an interval of 0.02. Resonant wavelength shift is calculated by Δ*λ* = *λ*(*ε_r_*,*t*) – *λ*(1,*t*). For the microring sensor (*t* = 0), the sensitivity is only 5.9 nm/RIU. For the NPM sensor, the sensitivity increases with NPM layer thickness. When the thickness of the NPM layer is 0.06 μm, 0.09 μm, 0.12 μm, and 0.15 μm, the corresponding sensitivity will be 28.4 nm/RIU, 64.2 nm/RIU, 136.8 nm/RIU, and 240.7 nm/RIU, respectively. Therefore, the essence for the enhancement of sensitivity is the evanescent wave amplified by the metamaterial. Interestingly, we find that the sensitivity of the NPM sensor can be up to 327.3 nm/RIU when NPM thickness is 0.174 μm. But when the thickness is larger than 0.174 μm, WGM with m = 27 will be transferred to the WGM with m = 26 or 28. Details are not shown here for brevity.

**Figure 9.**
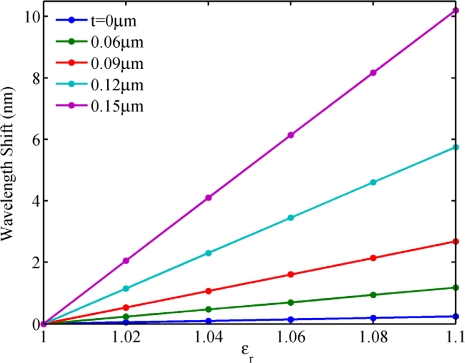
Surface sensing. Relation between *ε_r_* and wavelength shift for a variation of NPM layer thickness.

Surface sensing performance of the NPM sensor can also be analyzed according to the above procedures, and it is not shown here for brevity. Figure 9 shows the simulation results for surface sensing. Similarly, the sensitivity increases with NPM layer thickness. When the thickness of the NPM layer is 0.06 μm, 0.09 μm, 0.12 μm, and 0.15 μm, the sensitivity of the NPM sensor will be 24.1 nm/RIU, 54.9 nm/RIU, 117.7 nm/RIU, 208.9 nm/RIU, respectively. Therefore, sensitivity of the NPM sensor can be greatly improved by increasing the thickness of the NPM layer attached to its inner side. This is a novel method for sensor design with specified sensitivity.

## 4. Conclusions

WGMs of a dielectric waveguide with a layer of negative permeability metamaterial are theoretically analyzed, and the dispersion relation is derived. Analytical results of the resonant frequency shift and electric field distribution of the sensor are in good agreement with the simulation results. We show that the NPM sensor possesses a higher sensitivity than the traditional microring sensor, due to the amplification of the evanescent wave. Moreover, the sensitivity will be further improved by increasing the thickness of the metamaterial layer, opening a door for the design of novel sensors with desired sensitivity.

## Figures and Tables

**Table 1.** Comparison of the analytical frequency and simulated frequency for the microring sensor and the NPM sensor.

**Mode (*m*)**	**25**	**26**	**27**	**28**	**29**
**Theoretical results for *t* = 0 *μ*m (THz)**	186.145	192.199	198.251	204.300	210.347
**Simulation results for *t* = 0 *μ*m (THz)**	186.156	192.208	198.257	204.304	210.351
**Deviation (THz)**	0.011	0.009	0.006	0.004	0.004
**Theoretical results for *t* = 0.12 *μ*m (THz)**	180.484	186.179	191.844	197.476	203.072
**Simulation results for *t* = 0.12 *μ*m (THz)**	180.493	186.186	191.850	197.481	203.076
**Deviation (THz)**	0.009	0.007	0.006	0.005	0.004

## References

[1] Pendry JB (2000). Negative refraction makes a perfect lens. Phys. Rev. Lett.

[2] Pendry JB, Schurig D, Smith DR (2006). Controlling electromagnetic fields. Science.

[3] Alitalo P, Tretyakov S (2009). Electromagnetic cloaking with metamaterials. Mater. Today.

[4] Jiang WX, Chin JY, Cui TJ (2009). Anisotropic metamaterial devices. Mater. Today.

[5] Dubinov AE, Mytareva LA (2010). Invisible cloaking of material bodies using the wave flow method. Phys. Usp.

[6] Yang JJ, Huang M, Yang CF, Xiao Z, Peng JH (2009). Metamaterial electromagnetic concentrators with arbitrary geometries. Opt. Express.

[7] Jakšić Z, Djurić Z, Kment C (2007). A consideration of the use of metamaterials for sensing applications: Field fluctuations and ultimate performance. J. Opt. A.

[8] He S, Jin Y, Ruan ZC, Kuang JG (2005). On subwavelength and open resonators involving metamaterials of negative refraction index. New J. Phys.

[9] Melik R, Unal E, Perkgoz NK, Puttlitz C, Demir HV (2010). Metamaterial based telemetric strain sensing in different materials. Opt. Express.

[10] Lee HJ, Yook JG (2008). Biosensing using split-ring resonators at microwave regime. Appl. Phys. Lett.

[11] Cubukcu E, Zhang S, Park YS, Bartal G, Zhang X (2009). Split ring resonator sensors for infrared detection of single molecular monolayers. Appl. Phys. Lett.

[12] Alù A, Engheta N (2008). Dielectric sensing in ε-near-zero narrow waveguide channels. Phys. Rev. B.

[13] Shreiber D, Gupta M, Cravey R (2010). Comparative study of 1-D and 2-D metamaterial lens for microwave nondestructive evaluation of dielectric materials. Sens. Actuat. A.

[14] Zheludev NI (2010). The road ahead for metamaterials. Science.

[15] Huang M, Yang JJ, Sun J, Shi JH, Peng JH (2009). Modelling and analysis of Ω-shaped double negative material-assisted microwave sensor. J. Infrared Millimeter Terahertz Waves.

[16] Yang JJ, Huang M, Xiao Z, Peng JH (2010). Simulation and analysis of asymmetric metamaterial resonator-assisted microwave sensor. Mod. Phys. Lett. B.

[17] Huang M, Yang JJ, Petrin A (2011). Microwave sensor using metamaterials. Wave Propagation.

[18] White IM, Oveys H, Fan XD (2006). Liquid-core optical ring-resonator sensors. Opt. Lett.

[19] Walther C, Scalari G, Amanti MI, Beck M, Faist J (2010). Microcavity laser oscillating in a circuit-based resonator. Science.

[20] Ladd TD, Jelezko F, Laflamme R, Nakamura Y, Monroe C, O’Brien JL (2010). Quantum computers. Nature.

[21] Vahala KJ (2003). Optical microcavities. Nature.

[22] Armani DK, Kippenberg TJ, Spillane SM, Vahala KJ (2003). Ultra-high-Q toroid microcavity on a chip. Nature.

[23] Hunt HK, Soteropulos C, Armani AM (2010). bioconjugation strategies for microtoroidal optical resonators. Sensors.

[24] Vollmer F, Arnold S (2008). Wispering-gallery-mode biosensing: Labelfree detection down to single molecules. Nat. Methods.

[25] Zhu J, Ozdemir SK, Xiao YF, Li L, He L, Chen DR, Yang L (2010). On-chip single nanoparticle detection and sizing by mode splitting in an ultrahigh-Q microresonator. Nat. Photonics.

[26] Armani AM, Kulkarni RP, Fraser SE, Flagan RC, Vahala KJ (2007). Label-free, single-molecule detection with optical microcavities. Science.

[27] Ma Q, Rossmann T, Guo Z (2010). Whispering-gallery mode silica microsensors for cryogenic to room temperature measurement. Meas. Sci. Technol.

[28] Passaro VMN, Dell’Olio F, de Leonardis F (2007). Ammonia optical sensing by microring resonators. Sensors.

[29] Orghici R, Lützow P, Burgmeier J, Koch J, Heidrich H, Schade W, Welschoff N, Waldvogel SA (2010). Microring resonator sensor for sensitive detection of 1,3,5-Trinitrotoluene (TNT). Sensors.

[30] Yeh C, Shimabukuro F (2008). The Essence of Dielectric Waveguides.

[31] Heebner J, Grover R, Ibrahim T, Ibrahim T (2008). Optical Microresonators: Theory, Fabrication, and Applications.

